# Aorto-Right Atrial Fistula Resulting From a Ruptured Right Coronary Sinus of Valsalva Aneurysm

**DOI:** 10.7759/cureus.104490

**Published:** 2026-03-01

**Authors:** Chandler Pugh, Kristina Snoddy, Jamey Hammock, Adam Witcher

**Affiliations:** 1 Medicine, Edward Via College of Osteopathic Medicine, Auburn, USA; 2 Cardiology, University of Alabama at Birmingham St. Vincent's East, Birmingham, USA; 3 Cardiothoracic Surgery, University of Alabama at Birmingham St. Vincent's East, Birmingham, USA

**Keywords:** cardiac fistula, cardiac shunt, ruptured sinus of valsalva, sinus of valsalva aneurysm (sova), surgical case report

## Abstract

A sinus of Valsalva aneurysm (SOVA) is a rare cardiac anomaly characterized by dilation of one of the aortic sinuses, most commonly the right coronary or noncoronary sinus. A SOVA can be clinically silent or present acutely when rupture occurs or more gradually with progressive left-to-right shunting and high-output heart failure. Etiologies of SOVA include congenital failure of the aortic media and annulus to fuse, or an acquired SOVA from endocarditis, trauma, or connective tissue disorders. SOVAs are typically diagnosed by transthoracic echocardiography (TTE), and most patients undergo a surgical approach to treatment.

We report a case of a 54-year-old female who presented with chest pain, dyspnea, palpitations, and epistaxis, and was ultimately diagnosed with a ruptured right coronary SOVA forming a fistulous connection to the right atrium. Chest pain, dyspnea, and palpitations were attributed to the ruptured SOVA, whereas the epistaxis was considered incidental. Subsequent transesophageal echocardiography (TEE) revealed a ruptured right coronary SOVA forming a fistulous connection to the right atrium, producing a significant continuous left-to-right shunt with a calculated Qp/Qs of 2.6. She underwent median sternotomy with closure of the aneurysm on the aortic side and additional atrial ligation using a Dacron patch. This case is notable for the patient's atypical demographic profile, the absence of associated anomalies, and the presence of a systolic ejection murmur. It also highlights diagnostic limitations of TTE in this condition, as visualization may be hindered by suboptimal acoustic windows and the complex anatomy of sinus-to-atrial fistulous tracts. Early use of TEE can therefore be critical for accurate diagnosis and timely surgical intervention to prevent hemodynamic deterioration.

## Introduction

A sinus of Valsalva aneurysm (SOVA) is a rare cardiac anomaly characterized by dilation of one of the aortic sinuses, most commonly the right coronary or noncoronary sinus [1]. The prevalence of SOVA is approximately 0.09% among patients undergoing cardiac surgery. Despite their rarity, SOVAs are clinically significant because they may remain asymptomatic for years or can present abruptly with life-threatening rupture [1,2]. Ruptured SOVAs predominantly affect younger patients, with reported mean ages ranging from 32 to 39 years, and demonstrate a male predominance with male-to-female ratios of 2.2-3:1 [3,4]. They are frequently associated with congenital cardiac abnormalities, with ventricular septal defects present in approximately 50% of cases and aortic regurgitation in approximately 33% [5-7].

Typical rupture patterns follow predictable anatomic pathways. The right coronary sinus is the most common origin (80-88% of cases), followed by the noncoronary sinus (16-20%), with left coronary sinus involvement being rare (<1%). Rupture occurs predominantly into right-sided cardiac chambers: the right ventricle in 58-84% of cases and the right atrium in 27-41% [2,4,8-10]. Rupture into left-sided chambers or the pericardium is exceedingly rare [2,9].

Congenital versus acquired SOVAs differ in patient demographics and imaging characteristics. Congenital SOVAs, which account for approximately 82% of cases, result from failure of fusion between the aortic media and the annulus fibrosus, predisposing the sinus wall to progressive weakening and dilation [11,12]. These typically present in younger patients (mean age of 32-37 years) and are frequently associated with other congenital cardiac abnormalities, particularly supracristal ventricular septal defects (50-59% of cases) and bicuspid aortic valve [2,4,10].

Acquired SOVAs, comprising approximately 18% of cases, occur secondarily to conditions affecting the aortic wall, including infective endocarditis, chest trauma, or connective tissue disorders such as Marfan syndrome or Ehlers-Danlos syndrome [2,11,13]. These are typically present in older patients and are less commonly associated with other congenital cardiac anomalies [5,8,11-13]. Imaging clues suggesting acquired etiology include thickened, irregular aneurysm walls, evidence of aortic valve vegetations or destruction (in endocarditis), or generalized aortopathy (in connective tissue disorders) [11,13-16].

Clinical presentation varies depending on whether the SOVA is unruptured or ruptured. Symptoms arise when progressive enlargement causes mass effect, valvular distortion, or rupture into an adjacent cardiac chamber [1]. Ruptured SOVAs typically create a fistulous connection to right-sided cardiac chambers, producing a left-to-right shunt that may lead to volume overload, pulmonary hypertension, and high-output heart failure [4,8,17]. On physical examination, a continuous "machinery" murmur is the classic finding, present in approximately 76% of cases of ruptured SOVA [9]. Ruptured SOVAs constitute a surgical emergency, but the anatomy and shunt severity must first be determined before proceeding to intervention [18]. Contemporary diagnostic approach for ruptured SOVA relies primarily on echocardiography [13,19-21]. Transthoracic echocardiography (TTE) is the preferred initial diagnostic tool and can reliably diagnose most SOVAs [13,20,21]. When TTE is nondiagnostic or cannot adequately define the aneurysm's origin, course, or relationship to adjacent structures, transesophageal echocardiography (TEE) should be performed for confirmation [13,20,21]. Advanced cross-sectional imaging with cardiac CT or MRI provides valuable complementary anatomic information and is increasingly utilized for comprehensive preoperative planning [13,14,20]. Cardiac catheterization, while historically routine, is now optional in contemporary practice when echocardiography and cross-sectional imaging provide adequate anatomic detail [13,19,20]. The diagnosis is supported by visualization of intracardiac shunting and identification of an oxygen saturation “step-up” within the right-sided chambers during catheterization [11]. Catheterization may still be considered when additional hemodynamic assessment is needed or when coronary anatomy must be defined in older patients [13,14,19]. Definitive management involves closure of the aneurysmal defect and correction of any associated structural abnormalities [17]. Surgical repair, most commonly via patch closure, remains the standard treatment, particularly for patients with significant aortic regurgitation or complex associated lesions [22,23]. Transcatheter closure has emerged as an alternative in selected patients with small-sized ruptures, mild or no aortic regurgitation, and absence of complex associated abnormalities requiring surgical correction [22-24].

Here, we present a case of a 54-year-old female with a ruptured right coronary SOVA with a fistulous communication with the right atrium. This case is notable for the patient's atypical demographic profile, the absence of associated anomalies, and the presence of a systolic ejection murmur rather than the typical continuous machinery murmur. Her case demonstrates the value of prompt evaluation with TEE in suspected SOVA that cannot be definitively identified on TTE, and the favorable outcomes achievable with prompt surgical intervention.

## Case presentation

A 54-year-old female presented to the emergency department with angina, dyspnea, palpitations, and incidental epistaxis. Initial evaluation revealed tachycardia, cardiomegaly, and a 3/6 systolic ejection murmur at the right upper sternal border.

Initial TTE findings (obtained four weeks prior; Videos 1, 2) demonstrated a small, round structure measuring approximately 1.7 cm on the ventricular side of the tricuspid valve with pulsatile flow, with a differential diagnosis of unruptured SOVA or perimembranous ventricular septal defect. At that time, she was recommended for TEE, as TTE demonstrated insufficient definition to make a diagnosis. However, she declined admission for personal reasons. Her symptoms at that time were consistent with New York Heart Association (NYHA) class II heart failure, with dyspnea only on exertion. She returned to the emergency department four weeks later due to acute worsening of her dyspnea and angina to NYHA class IV symptoms, with profound angina, dyspnea, palpitations, and incidental epistaxis.

**Video 1:** Two-dimensional transthoracic echocardiography (TTE). TTE revealed a small, round structure on the ventricular side of the tricuspid valve, with a pulsatile flow. A differential diagnosis of an unruptured sinus of Valsalva aneurysm or a perimembranous ventricular septal defect was established.

**Video 2:** Two-dimensional transthoracic echocardiography (TTE) with color Doppler. TTE revealed a small, round structure on the ventricular side of the tricuspid valve, with a pulsatile flow. A differential diagnosis of an unruptured sinus of Valsalva aneurysm or a perimembranous ventricular septal defect was established.

Subsequent TEE findings (Videos 3, 4) revealed a ruptured right coronary SOVA measuring 3.3 cm, representing significant interval enlargement from the prior TTE. The aneurysm had formed an aortico-right atrial fistula, resulting in a continuous left-to-right shunt with a calculated Qp/Qs of 2.6 (normal <1.0; values >2.0 indicate a large, hemodynamically significant shunt). The superior visualization and larger measured size on TEE compared to TTE established the definitive diagnosis and indicated the need for urgent intervention.

**Video 3:** Two-dimensional transesophageal echocardiography (TEE). TEE demonstrates a sinus of Valsalva aneurysm of the right coronary sinus with a fistulous connection to the right atrium.

**Video 4:** Two-dimensional transesophageal echocardiography (TEE) with color Doppler. TEE demonstrates a sinus of Valsalva aneurysm of the right coronary sinus with a fistulous connection to the right atrium.

Cardiac catheterization was performed to confirm hemodynamic significance and assess for associated coronary disease, with findings depicted in Table 1. Right heart catheterization demonstrated elevated right-sided filling pressures consistent with volume overload from the left-to-right shunt: right atrial pressure = 18 mmHg (normal = 0-5 mmHg), right ventricular pressure = 44/14 mmHg (normal = 15-30/0-8 mmHg), pulmonary artery pressure = 39/25 mmHg (normal = 15-30/4-12 mmHg), and pulmonary capillary wedge pressure = 20 mmHg (normal = 6-12 mmHg). Oximetry revealed a step-up at the right atrium, confirming a significant left-to-right shunt. Cardiac output was preserved. Left heart catheterization via the radial artery demonstrated an aortic pressure of 102/59 mmHg and a left ventricular end-diastolic pressure of 21 mmHg (normal <12 mmHg). Coronary angiography revealed normal coronary arteries. Aortography confirmed abnormal flow originating from the right coronary sinus, consistent with the TEE findings. The hemodynamic data confirmed a large, hemodynamically significant shunt (Qp/Qs >2.0) with biventricular volume overload and elevated filling pressures, meeting clear indications for surgical repair.

**Table 1:** Diagnostic findings from echocardiography and cardiac catheterization. TTE: transthoracic echocardiography; TEE: transesophageal echocardiography; RV: right ventricle; LV: left ventricle.

Hemodynamic measure	Result	Reference	Interpretation
Initial aneurysm size (TTE)	1.7 cm	N/A	Initial measurement
Subsequent aneurysm size (TEE)	3.3 cm	N/A	Interval enlargement; ruptured
Qp/Qs ratio	2.6	1.0	Large left-to-right shunt; surgical indication
Right atrial pressure	18 mmHg	0-5 mmHg	Elevated; volume overload
Right ventricular pressure	44/14 mmHg	15-30/0-8 mmHg	Elevated; RV volume overload
Pulmonary artery pressure	39/25 mmHg	15-30/4-12 mmHg	Elevated; secondary to shunt
Pulmonary capillary wedge pressure	20 mmHg	6-12 mmHg	Elevated; left-sided congestion
Left ventricular end-diastolic pressure	21 mmHg	12 mmHg	Elevated; LV volume overload
Aortic pressure	102/59 mmHg	90-140/60-90 mmHg	Normal systolic; wide pulse pressure

The patient underwent successful surgical repair via median sternotomy with cardiopulmonary bypass. The aneurysm was closed on the aortic side using a Dacron patch with additional atrial ligation. Intraoperative TEE demonstrated exclusion of the aneurysmal sac with only trace residual aortic-to-right atrial flow. Intraoperative and postoperative coagulopathy required transfusion support; however, the remainder of her postoperative course was uncomplicated. By postoperative day five, the patient was ambulating independently and was discharged home. At the 30-day follow-up, she demonstrated complete resolution of heart failure symptoms and full functional recovery.

## Discussion

A SOVA is an uncommon but clinically significant cardiac abnormality that can lead to severe hemodynamic compromise if it ruptures. The consequences of a ruptured SOVA depend primarily on the size and acuity of the rupture. Large, abrupt ruptures can cause rapid right-sided volume overload, hepatomegaly, peripheral edema, and respiratory distress, while smaller or more gradual ruptures may initially be subtle, with dyspnea and angina [11]. Over time, chronic volume overload can lead to chamber dilation, tricuspid regurgitation, and a progressive decline in functional capacity [20].

Imaging and operative decision-making

TTE is typically the first-line imaging modality for SOVA and is generally diagnostic, with reported sensitivity of 93.9% and accuracy of 99.8%. In a study of 212 SOVAs, 13 were missed by TTE, with 77% being small aneurysms of the right coronary sinus extending into the right ventricle across a ventricular septal defect (VSD) [7]. In contrast, our case involved an unruptured SOVA from the right coronary sinus without a VSD that was appropriately detected on initial TTE as a 1.7 cm structure near the tricuspid valve with pulsatile flow. However, TTE could not clearly define the structure’s origin or its relationship to the aortic root and adjacent cardiac chambers, likely due to acoustic window limitations, and the differential diagnosis, therefore, included both an unruptured SOVA and a perimembranous VSD. Because of this limited anatomic definition, TEE was recommended for further characterization; however, the patient declined admission at that time.

When she returned four weeks later with acute clinical deterioration to NYHA class IV, TEE revealed interval rupture with aneurysm enlargement to 3.3 cm and formation of an aortico-right atrial fistula. Aortico-right atrial fistulas represent approximately 27% of ruptured SOVAs and are the second most common rupture site after the right ventricle [4,7]. TEE provides high-resolution images of the thoracic aorta and is particularly useful in detailing aortic valve anatomy and fistulous communications, which were critical for surgical planning in this case. This case illustrates the natural history of untreated SOVA, with progression from an unruptured aneurysm to acute rupture over a relatively short interval, underscoring the importance of timely intervention once the diagnosis is established.

When TTE is nondiagnostic and clinical suspicion remains high based on persistent findings (continuous murmur, right-sided volume overload), TEE should be performed promptly. TEE provides superior visualization of the aortic root and can precisely delineate the origin, course, and termination of the aneurysm [21,25]. Alternative cross-sectional imaging modalities, including cardiac computed tomography angiography (CTA) and cardiac magnetic resonance (CMR), provide excellent anatomic detail and comprehensive assessment [13,20,26]. CTA offers rapid acquisition with high spatial resolution, while CMR enables comprehensive assessment of anatomy, function, and flow quantification without ionizing radiation [13-15]. In this case, TEE was favored because it could be performed rapidly at the bedside in a hemodynamically unstable patient, provided real-time dynamic assessment, and allowed immediate procedural planning.

Surgical intervention remains the standard of care for both ruptured and intact SOVAs, particularly in patients with associated cardiac lesions, significant aortic regurgitation, or complex anatomy [22,27,28]. The most common approach is patch closure of the defect from the aortic side using synthetic materials such as Dacron. When performed promptly, outcomes are generally excellent, with rapid symptomatic improvement and reversal of high-output heart failure physiology. Potential complications include bleeding, aortic insufficiency, arrhythmias, or residual shunts [26,27]. Transcatheter closure has emerged as an alternative in appropriately selected patients. Percutaneous closure is most suitable for patients with small-to-moderate-sized ruptures (typically 10-12 mm at the aortic end), no or mild aortic regurgitation, and absence of complex associated lesions such as large VSDs, bicuspid aortic valve, or endocarditis [22-24]. These findings are most applicable to hemodynamically stable patients with isolated, small-to-moderate ruptured SOVAs; in unstable patients or those with complex anatomy, surgery remains the preferred approach. In this case, surgical repair was favored due to the large, high-flow aorto-right atrial fistula (Qp/Qs of 2.6) with significant hemodynamic compromise and rapid interval enlargement of the ruptured SOVA, as these features are associated with a higher risk of device instability and residual shunting with percutaneous closure [22,27,28].

Long-term surveillance

Following surgical repair, long-term surveillance is essential to monitor for residual or recurrent shunting, progressive aortic regurgitation, and prosthetic valve dysfunction [4,29]. In a 30-year surgical series, freedom from moderate or severe aortic regurgitation was 98.5% at 10 years, with actual survival of 99% at 10 years [4]. Recommended follow-up includes clinical assessment and transthoracic echocardiography at one, three, six, and 12 months postoperatively, then annually thereafter to monitor for aneurysm recurrence and aortic valve dysfunction [30].

Clinical implications

This case highlights the diagnostic challenges associated with SOVA, especially when initial imaging is inconclusive. It demonstrates the importance of rapid recognition and early surgical management to prevent rupture. This patient's near-complete recovery at 30 days illustrates the favorable prognosis achievable with prompt management and reinforces the need to consider SOVA in patients presenting with new continuous murmurs, unexplained right-sided volume overload, or evidence of acute left-to-right shunting.

## Conclusions

Ruptured SOVA, though rare, should be considered in patients with unexplained murmurs, shunt physiology, or acute right heart failure symptoms. TTE is often the initial study and is typically sufficient for diagnosis; however, if unrevealing, TEE and angiography should be considered. Dacron patch repair is an excellent option for ruptured SOVA, as demonstrated in this patient’s reversal of heart failure symptomology.
